# Teething Syrups and Powders

**Published:** 1870-02

**Authors:** 


*Teething Syrups and Powders.*—A correspondent of the *California Medical Gazettee* writes that he was called to an infant of six months old, who was in a dying condition, apparently from the effects of a narcotic poison. It had taken no medicine except "Mrs. Winslow's Soothing Syrup," of which it had taken within ten hours, two doses of about one teaspoonful each. A skillful chemist analysed the syrup remaining in the bottle, about 10 drachms, and it yielded of morphine and other opium alkaloids 1 14-100 grains, very nearly one grain to the ounce of syrup. Following the directions upon the label there is a dose of morphine equal to 10 drops of laudanum, given to a child of three months old every two hours, and double the quantity to a child of six months old. As four drops of laudanum have been known to kill an infant of nine months, this writer thinks that the analysis of this syrup should be published, that the profession, and through it the public may be warned of the fearful effects of administering this dangerous and popular nostrum.

In the *London Med. Times and Gazette* appears an analysis of "Stedman's Teething powder," an English nostrum, as follows : Calomel, 42.03 ; Organic Matter, 57.97 ; Ash, trace ; in all 100.00 The organic matter appeared to be quite inert. It was soluble in water, giving a solution having no particular taste. No opium was detected. There was no corrosive sublimate. These powders vary in weight from 1.42 grain up to 2.78 grain, and each one contains from about 6-10 grain up to 1 1-10 grain of Calomel mixed with rather more than its own weight of some diluent substance.

